# Thallium Labeled Citrate-Coated Prussian Blue Nanoparticles as Potential Imaging Agent

**DOI:** 10.1155/2018/2023604

**Published:** 2018-04-26

**Authors:** Krisztián Szigeti, Nikolett Hegedűs, Kitti Rácz, Ildikó Horváth, Dániel S. Veres, Dávid Szöllősi, Ildikó Futó, Károly Módos, Tamás Bozó, Kinga Karlinger, Noémi Kovács, Zoltán Varga, Magor Babos, Ferenc Budán, Parasuraman Padmanabhan, Balázs Gulyás, Domokos Máthé

**Affiliations:** ^1^Department of Biophysics and Radiation Biology, Semmelweis University, Budapest 1094, Hungary; ^2^Department of Radiology and Oncotherapy, Semmelweis University, Budapest 1094, Hungary; ^3^CROmed Translational Research Centers, Budapest 1047, Hungary; ^4^Institute of Materials and Environmental Chemistry, Research Center for Natural Sciences of the Hungarian Academy of Sciences, Budapest, Hungary; ^5^Mediso Medical Imaging Systems Ltd., Budapest 1022, Hungary; ^6^Department of Public Health Medicine, Medical School, University of Pécs, Pécs 7624, Hungary; ^7^Lee Kong Chian School of Medicine, Nanyang Technological University, Singapore; ^8^Imperial College London, Kensington, London, UK

## Abstract

**Background:**

The aim of this study was to develop and characterize a nanoparticle-based image-contrast platform which is biocompatible, chemically stable, and accessible for radiolabeling with ^201^Tl. We explored whether this nanoparticle enhanced the T1 signal which might make it an MRI contrast agent as well.

**Methods:**

The physical properties of citrate-coated Prussian blue nanoparticles (PBNPs) (iron(II);iron(III);octadecacyanide) doped with ^201^Tl isotope were characterized with atomic force microscopy, dynamic light scattering, and zeta potential measurement. PBNP biodistribution was determined by using SPECT and MRI following intravenous administration into C57BL6 mice. Activity concentrations (MBq/cm^3^) were calculated from the SPECT scans for each dedicated volume of interest (VOI) of liver, kidneys, salivary glands, heart, lungs, and brain.

**Results:**

PBNP accumulation peaked at 2 hours after injection predominantly in the kidneys and the liver followed by a gradual decrease in activity in later time points.

**Conclusion:**

We synthetized, characterized, and radiolabeled a Prussian blue-based nanoparticle platform for contrast material applications. Its in vivo radiochemical stability and biodistribution open up the way for further diagnostic applications.

## 1. Introduction

Two or more imaging techniques applied simultaneously or within a very close timeframe ideally complement each others. Powerful new high-resolution imaging tools such as optical whole body imaging, micro-X-ray-based computed tomography (CT), and magnetic resonance imaging (MRI) could thus nowadays enable very good anatomical detail fusion to functional image data of living organisms and disease. These functional data have been obtained for decades in nuclear medicine using positron emission tomography (PET) and single photon emission computed tomography (SPECT). PET and SPECT possess unparalleled sensitivity and functionality but their resolution can be enhanced much further by hybrid imaging. This provides the ability of acquiring (almost) simultaneous in vivo images of highly resolved anatomy and accurately measured physiology in a noninvasive manner in animal models, clinical research, or diagnostics. This approach enables the explicit localization as well as the quantification of metabolic activity in target tissues [1, 2]. Nanoparticles embody unique imaging possibilities. A high signal concentration for well-amplified biologic readouts can be combined by their use for both anatomy and (patho)physiology. With the availability of novel multimodal imaging devices, the demand for innovative multimodal contrast materials is higher than ever before [1, 3]. The greatest concern of in vivo functional imaging with isotopes is whether the isotope's binding to the nanoparticle is stable enough in the living organism. Magnetic resonance imaging (MRI) is an ideal modality to elucidate morphological qualities in living organisms. In the past two decades, research of MRI contrast agents such as nanoparticles (NP) with paramagnetic properties started to burgeon. These particles were mostly paramagnetic iron oxides such as magnetite (Fe_3_O_4_), maghemite (*γ*Fe_2_O_3_), and other ferrites. The contrast property of these materials (hypointense signal in T2-weighted images) is not optimal for anatomical mapping [4, 5], where, in turn, an enhancement (positive) in MRI signal is expected from contrast materials. To overcome these difficulties, the development of a proper T1 enhancing iron-containing particulate contrast agent seemed to be a good solution.

Our aim was to create a biocompatible Prussian blue nanoparticle (PBNP) (iron(II);iron(III);octadecacyanide) platform for preclinical imaging which has three important qualities: first, the long-term stability of the NP platform (surface characteristics, i.e., coating to avoid phagocytosis and aggregation, the stability of the NP component's diameter, and homogeneity and colloidal stability); second, T1 signal enhancement suitable for MR imaging; and third, the stability of the bond between the radionuclide and nanoparticle structure in most environments.

**Figure 1:**
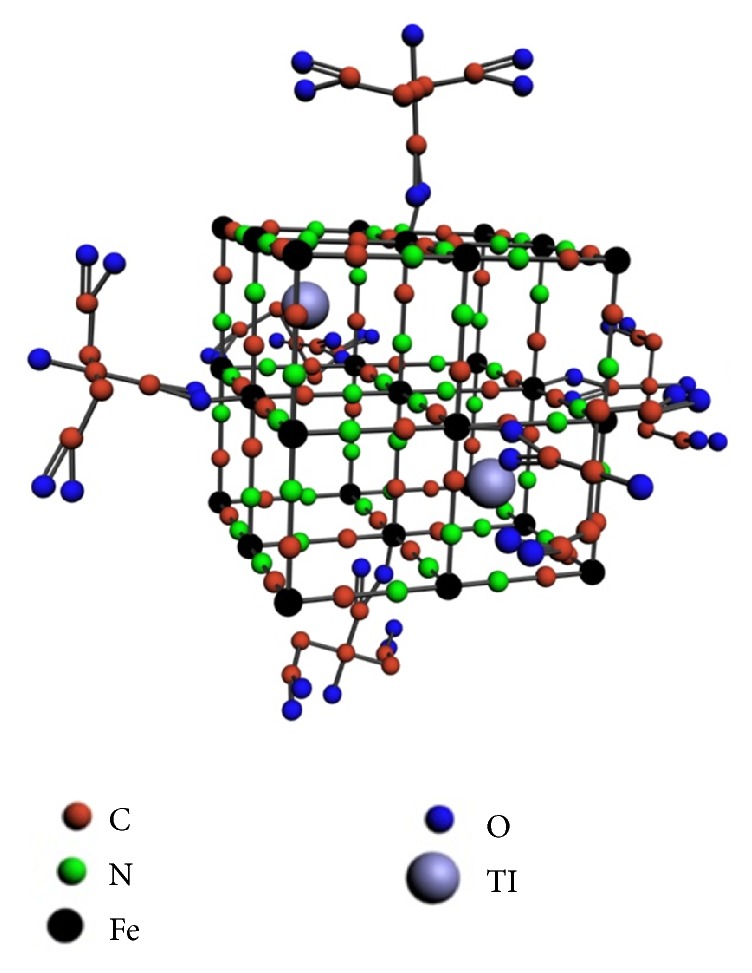
The structure of Prussian blue. Citrate bound to Fe(III) forms the coating. The colors represent the following ions or atoms, respectively: red: C; green: N; black: Fe (both of Fe(II) and Fe(III)); blue: O; gray: 201Tl.

Thanks to the special structure of PBNP (Figure 1), it is capable of very stably binding thallium cations and thereby acts as an appropriate SPECT radiotracer [6, 7].

The exact binding mechanism has not been published until this time. In general chemical ion-exchange, physical adsorption (Prussian blue crystals are dubbed “chemical sponges” to absorb metal ions [4, 8]), and ion trapping may all be involved in the PBNP-thallium binding process depending on the pH condition and physical properties of particles (e.g., particle size or moisture content) [6]. The stability of the PBNP-thallium binding is high as quantified by Gupta which makes Prussian blue a suitable antidote for thallium poisoning [9].

The investigation of in vivo stability and biodistribution of the radiolabeled PBNPs was our goal as well. Quantitative tomographic imaging using emitted gamma rays was carried out in a dedicated small animal SPECT/CT instrument. Briefly, we wished to explore ^201^Tl radiolabeled PBNPs as a form of chemically stable contrast material with applications in MRI and SPECT. This approach has been patented in 2012 by two of the authors [10].

## 2. Materials and Methods

### 2.1. Synthesis of PBNPs

A modified method of Shokouhimehr was used to prepare citrate-coated PBNPs [4]. Briefly, PBNPs were prepared by slowly adding 20 mL of a 1.0 mM Fe(III) chloride (anhydrous) (FeCl_3_) (Sigma-Aldrich) solution containing 0.5 mmol of citric acid (Sigma-Aldrich) to a solution containing an equimolar amount of potassium ferrocyanide (anhydrous) (K_4_[Fe(CN)_6_]) (Sigma-Aldrich) under rigorous stirring at 40°C. Three samples (*n* = 3) were synthesized. The products were isolated by centrifugation at 29000 rpm (Beckman Ti 50.2 rotor, 30 min at 4°C) and then dialyzed for 3 × 1 hour (14 kDa filter) (Sigma-Aldrich, D9277).

### 2.2. In Vitro Measurements

#### 2.2.1. Atomic Force Microscopy

After 15 min incubation the mica surface was dried in N_2_ stream. AFM images were collected in noncontact mode with a Cypher S instrument (Asylum Research, Santa Barbara, CA) at 1 Hz line-scanning rate in air, using a silicon cantilever (OMCL AC-160TS, Olympus, Japan) oscillated at its resonance frequency (300–320 Hz). Temperature during the measurements was 29 ± 1°C. AFM amplitude-contrast images are shown in this paper. AFM images were analyzed by using the built-in algorithms of the AFM driver software (IgorPro, WaveMetrics Inc., Lake Oswego, OR). Particle statistics was done by analyzing a 10 × 10 *μ*m height-contrast image with *n* = 1162 particles. Maximum height values were taken as height of particles; rectangularity was calculated as the ratio of the particle area to the area of a nonrotated inscribing rectangle. The closer a particle is to a rectangle the closer this value is to unity.

#### 2.2.2. Transmission Electron Microscopy (TEM)

Morphological investigations of the NPs were carried out on a MORGAGNI 268(D) (FEI, Eindhoven, Netherlands) transmission electron microscope. Diluted sample was dropped and dried on a carbon-coated copper grid. Size distribution was determined by manually measuring the diameter of 700 particles on the images, using a software custom designed for this purpose (tem_circlefind by András Wacha, MTA TTK, Hungary).

#### 2.2.3. Zeta Potential and Light Scattering Measurement

Zeta potential and hydrodynamic diameter measurements were performed using a Zetasizer Nano ZS instrument (from Malvern Instruments Ltd., Worcestershire, UK), fitted with a He-Ne laser (*λ* = 633 nm) and a backscatter detector fixed at 173°. For zeta potential measurements, PBNP samples were diluted thirtyfold with a solution containing 0.9% NaCl in order to mimic physiological ion concentrations. The pH of the sample was adjusted by the addition of a 0.01 M NaOH solution. DLS measurement was performed in a W130i DLS instrument (Avid Nano Ltd., High Wycombe, UK) using low volume disposable cuvettes (UVette, Eppendorf Austria GmbH). The sample was diluted tenfold with ultrapure water and filtered through a 0.22 um membrane filter. Data were processed with iSize 2.0 software utilizing the CONTIN algorithm. DLS measurements were performed weekly for a period of 6 weeks to determine colloidal stability. Samples were stored at 4°C.

#### 2.2.4. Magnetic Resonance Imaging (MRI)

MRI measurements were performed in vitro with a nanoScan® PET/MR system (Mediso, Hungary) in a permanent magnetic field of 1 T in a 450 mT/m gradient system using a volume transmit/receive coil with a diameter of 60 mm. A phantom consisting of six 1.5 ml microcentrifuge tubes was prepared, with each microcentrifuge tube containing a 150 *μ*l aliquot of a PBNP solution. PBNP concentrations were 0.00 mM, 0.125 mM, 0.25 mM, 0.38 mM, 0.76 mM, and 1.25 mM. Particle concentrations were based on the molecular weight calculated using the illustrated crystal structure model (Figure 1). Four different sequences were used for imaging, two of them for relaxivity measurements and two others for basic imaging to exploit different contrast mechanisms. T1-weighted 2D Spin Echo sequence was acquired using the below parameters: field of view (FOV) 74 mm, 0.41 mm in-plane resolution, 1.6 mm slice thickness, and TR/TE 500/9.2 ms. Fast spin T2-weighted echo sequence was acquired with the same geometric parameters but with TR/TE 4000/77.1 ms and 4 averages. T1 relaxation rates and r1 relaxivity were calculated from inversion prepared snapshot gradient echo (IR GRE-SNAP) images acquired with 50 mm FOV, plane resolution of 0.39 mm, slice thickness of 2 mm, 8 averages, TR/snap TR/TE 12000/8.2/3 ms, flip angle of 8°, and 8 different transmission intervals ranging from 50 to 6400 ms. T2 relaxation rates and r2 relaxivity were calculated from multiecho spin echo (ME-SE) images scanned with 50 mm FOV, plane resolution of 0.5 mm, slice thickness of 2 mm, TR of 2000 ms, and 32 different TEs ranging from 15 to 480 ms. MRI signal enhancement of PBNPs was measured at six different concentrations (0.00 mM, 0.125 mM, 0.25 mM, 0.38 mM, 0.76 mM, and 1.25 mM). After scanning the concentration dependent signal changes were calculated and compared to the signal of pure water.

#### 2.2.5. Viability Assay

Human cervical carcinoma (HeLa) cells were chosen for cell viability assay tests. They were seeded at a density of 10^4^ cells/mL to each Petri dish. The cells were exposed to saline (negative control) and three different PBNP containing solutions (4-, 16-, and 32-fold dilutions of PBNP) for 20 min. The medium was then removed and the cells were rinsed in Hanks' Balanced Salt Solutions (HBSS; Sigma-Aldrich) twice. The rinsed cells were harvested by adding 200 *μ*l of 0.1% trypsin (Sigma-Aldrich) for 3 min, then adding 800 *μ*l of modified eagle medium (MEM; Sigma-Aldrich) with 0.1% trypan blue, and counting the number of living cells.

#### 2.2.6. Radiolabeling with ^201^Tl

Thallium-201labeled PBNPs were prepared as follows: 15 *μ*l (37 MBq) [^201^Tl]TlCl (Covidien, Netherlands) was added to 20 *μ*l PBNP in 250 *μ*l isotonic 0.9% NaCl solution (Braun, Hungary) and the reaction mixture was incubated at room temperature for 60 minutes. The calculated specific activity of the radiolabeled product was 62 GBq/g, based on the specific activity of [^201^Tl]TlCl, the size and molar weights of nonhydrated PBNPs, and the molar amount of Fe in the reaction vial.

#### 2.2.7. Chromatography of Radiolabeled NP Suspension

Radiochemical purity of the labeled suspension was determined by paper chromatography using 18 cm long Mackerey-Nagel 813 paper strips and double distilled water as mobile phase (*n* = 7). After photographic evaluation the chromatography paper was cut into 0.5 cm pieces and the activity of each piece was measured in a well-type NaI (Tl) scintillation detector (NZ-310 Gamma, Budapest). Retention factors (RF) were calculated as a ratio of the distance the spots and the solvent front had moved from the baseline.

### 2.3. In Vivo Measurements

#### 2.3.1. Animals

In vivo imaging was carried out in C57BL/6 male mice (*n* = 5 for SPECT/CT scans, *n* = 2 for MRI scans, Charles River, Hungary). Animals had ad libitum access to food and water and were housed under temperature-, humidity-, and light-controlled conditions. All procedures were conducted in accordance with the ARRIVE guidelines and the guidelines set by the European Communities Council Directive (86/609 EEC) and approved by the Animal Care and Use Committee of Semmelweis University (protocol number: XIV-I-001/29-7/2012). Mice were 10–12 weeks old with an average body weight of 28 ± 5 g. During imaging animals were kept under anesthesia using a mixture of 2.5% isoflurane gas and medical oxygen. Their body temperature was maintained at 37°C throughout imaging.

#### 2.3.2. SPECT/CT Imaging

Images were acquired with a NanoSPECT/CTPLUS (Mediso Ltd., Hungary) sequential animal SPECT/CT imaging system. For SPECT/CT imaging, 21.07 ± 2.38 MBq of ^201^Tl labeled PBNP was injected in 150 *μ*l physiological saline (Braun, Hungary) into a lateral tail vein.

During imaging animals were fixed to a MultiCellTM Imaging Chamber (Mediso Ltd., Hungary) to avoid movement and placed in the center of the FOV. Image acquisitions started with CT scanning (without any contrast agent) 60 min after the intravenous injection of ^201^Tl-labeled PBNP. Further SPECT/CT scans were acquired at 24, 48, and 72 hours after injection. Both the CT and subsequent SPECT imaging took 30 min each. The reconstructed cubic voxel side size was 150 *μ*m in a 198 × 198 × 546 voxel matrix in both the SPECT and CT modalities. Fusion (Mediso Ltd., Hungary) and VivoQuant (inviCRO LLC, US) image analysis software were used to further analyze the reconstructed, reoriented, and coregistered images by drawing appropriate volumes of interests (VOIs) over the specified target organs. These VOIs were delineated manually on each CT scan. Activity concentrations (MBq/cm^3^) were determined for each volume of interest and data was corrected for the scatter and isotopic decay during the reconstruction. Organs were initially ranked based on the visually representative uptake of activity. Then a quantitative threshold (0.1% of injected activity) was applied on the images. Organs with a higher uptake of 0.1% were quantified by placing three-dimensionally corresponding activity measuring VOIs on them. With this method the uptake of radioactivity was selected and measured in the following organs: liver, kidneys, salivary glands, heart, lungs, and brain.

#### 2.3.3. Magnetic Resonance Imaging (MRI)

Images were acquired before (prescan) and after the PBNP injection with a nanoScan® PET/MR system (Mediso, Hungary) in a permanent magnetic field of 1 T in a 450 mT/m gradient system using a volume transmit/receive coil with a diameter of 60 mm. For imaging 1.25 mM PBNP was injected in 250 *μ*l physiological saline (Braun, Hungary) into a lateral tail vein.

Two different sequences were used for imaging. T2-weighted 2D Fast Spin Echo sequence was acquired using the below parameters: field of view (FOV) 70 mm, 0.35 mm in-plane resolution, 0.2 mm slice thickness, and TR/TE 4809/70 ms. T1-weighted Gradiens-echo sequence was acquired using the below parameters: field of view (FOV) 70 mm, 0.40 mm in-plane resolution, 0.4 mm slice thickness, and TR/TE 3.4/25 ms.

## 3. Results

### 3.1. In Vitro Measurements

#### 3.1.1. Atomic Force Microscopy

**Figure 2:**
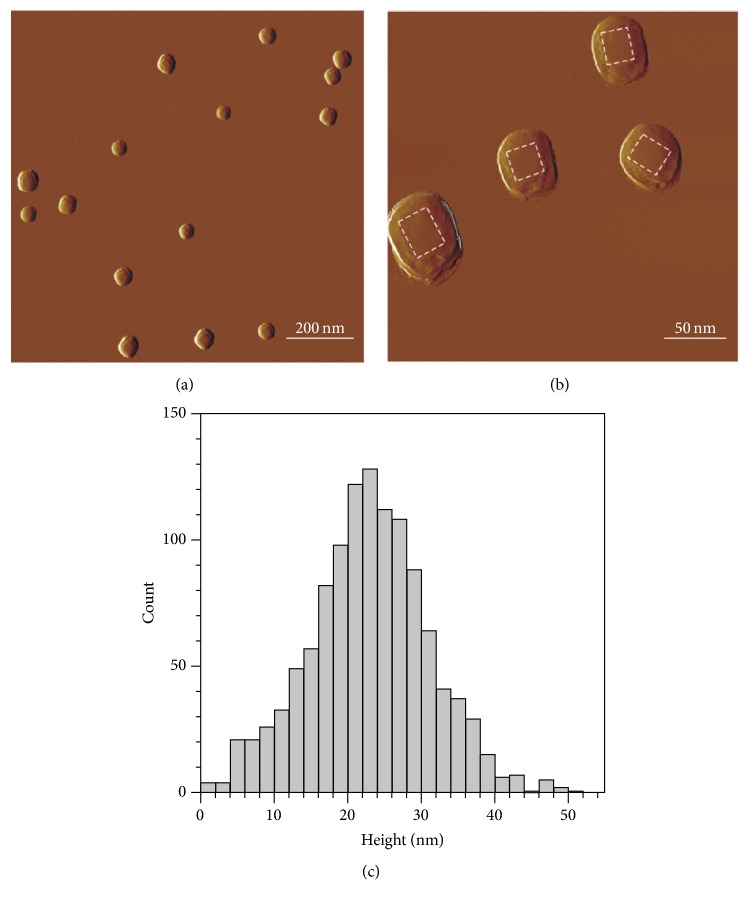
((a) and (b)) AFM amplitude-contrast images of PBNPs on mica surface. Contours of particles are shown by white dashed lines on (b). (c) Height distribution of PBNPs (*n* = 1162).

AFM images are shown in Figure 2. PBNPs appeared as objects with a flat rectangular surface protruding from a rounded halo (Figure 2(a)). The rectangular surface represents the real geometry of the particles (Figure 2(b)), while their halo is the consequence of tip convolution, that is, the effect of imaging a rectangular prism by a tetrahedral AFM tip. Rectangularity of the particles (together with their halo) was found to be 0.81 ± 0.09 (mean ± SD), indicating that PBNPs indeed represent rectangular topography. Height of the particles showed monomodal distribution with a mean ± SD of 23.0 ± 8.3 nm (Figure 2(c)).

#### 3.1.2. Transmission Electron Microscopy

**Figure 3:**
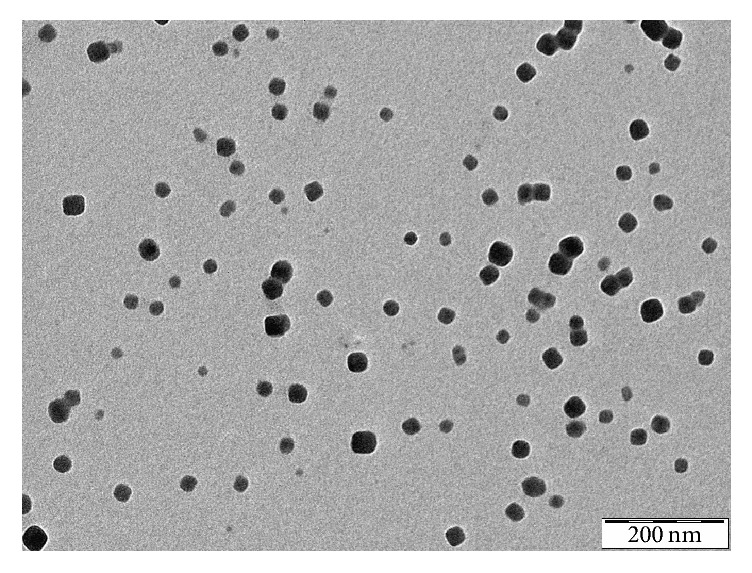
TEM image of PBNPs on carbon-coated copper grid.

The nonhydrated shape and size of the NPs were also analyzed with TEM. The shape of the nanoparticles on TEM and AFM images was similar. PBNPs appeared as flat rectangular, dense objects in this case as well. The mean diameter of the nanoparticles was 17.54 ± 4.56 nm (average ± SD, *n* = 700) (Figure 3).

#### 3.1.3. Zeta Potential and Light Scattering Measurement

The mean zeta potential of PBNPs at the measured pH range did not exceed 15 mV (*n* = 3). At pH 7.4 the zeta potential was −25.7 ± 1.8 mV (*n* = 3).

The mean hydrodynamic diameter (intensity-based harmonic mean or *z*-average) of citrate-coated PBNPs was 32.10 ± 0.1801 (average ± SD, *n* = 10), as determined by DLS. This had only changed slightly with time. There was no significant colloidal alteration during the 6-week duration of the study, as the calculated 0.203 ± 0.004 polydispersity index (PDI) shows the PBNPs did not flocculate or aggregate during this time (not illustrated).

#### 3.1.4. Magnetic Resonance Imaging

**Figure 4:**
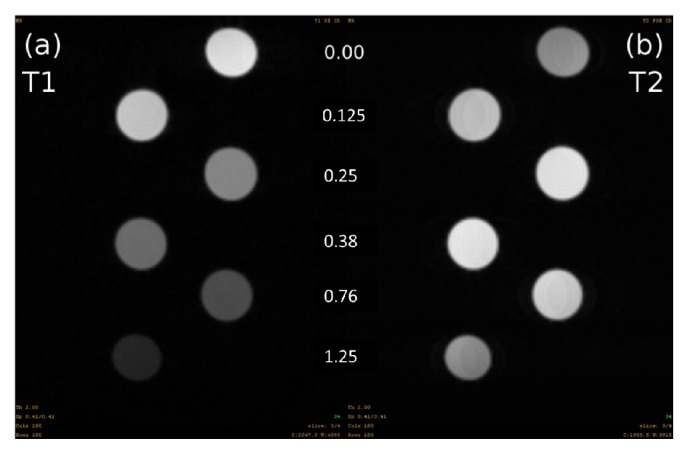
T1-weighted inversion prepared snapshot gradient echo (a) and T2-weighted multiecho spin echo (b) images of a phantom containing five different concentrations 0.125 mM, 0.25 mM, 0.38 mM, 0.76 mM, and 1.25 mM of PBNP solutions were scanned and signal changes compared to the bidistilled water signal.

T1-weighted (Figure 4(a)) and T2-weighted spin echo (Figure 4(b)) images of a phantom (containing five different concentrations (0.00 mM, 0.125 mM, 0.25 mM, 0.38 mM, 0.76 mM, and 1.25 mM) of PBNP solutions and a pure solvent) were scanned to visually evaluate the signal enhancement on T1-weighted image instead of decreased signal on T2-weighted image. Based on the inversion prepared gradient echo scan and the multislice multiecho scan T1 and T2 relaxation rates were calculated. Afterward from these values, longitudinal relaxivity (*r*1 = 0.64 ± 0.02 mM^−1 ^ms^−1^) and transversal relaxivity values (*r*2 = 0.96 ± 0.03 mM^−1 ^ms^−1^) were fitted and are presented in Table 1.

#### 3.1.5. Viability Assay

The survival rate of HeLa cells exposed to PBNP was above 95%. There was almost no difference between the numbers of intact cells in case of different PBNP concentration. For detailed information, see Table S1.

#### 3.1.6. Stability of Radiolabeling

Using paper chromatography, we found that both unlabeled and radiolabeled particles have an Rf value of 0.0–0.1. Nonreacted ^201^Tl ions moved together with the solvent front. Only 2% of the whole ^201^Tl activity was detected at the solvent front. Therefore, the radiochemical purity was estimated as being >98% in all experiments (Table 2). Comparing the total added activity of ^201^Tl to the solution and particle-bound ^201^Tl activity the radioactive labeling yield was 99.84% (SD: 1.01%).

### 3.2. In Vivo Biodistribution Studies

#### 3.2.1. SPECT/CT Imaging

**Figure 5:**
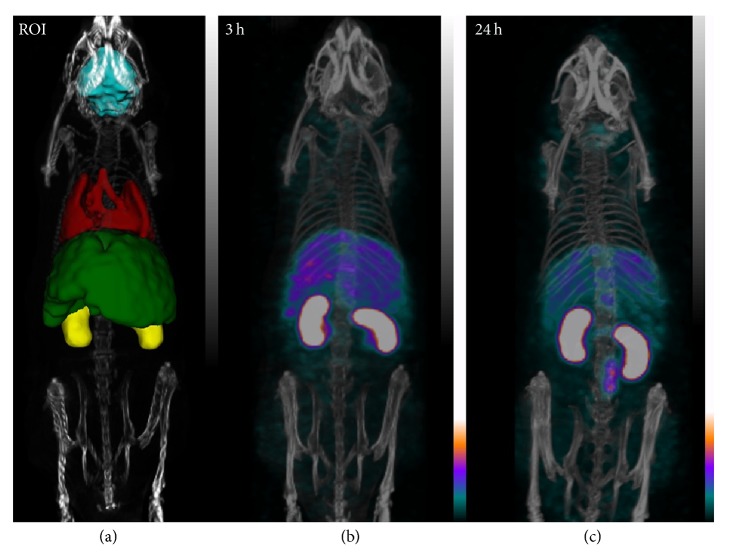
VOI of liver, kidneys, heart, and brain (a). Biodistribution of ^201^Tl labeled PBNPs after 2 hours (b) and 24 hours (c) injection. The mice were under isoflurane anesthesia during the 30 minutes long SPECT scans. The injected activity was 21.07 ± 2.38 MBq.

**Figure 6:**
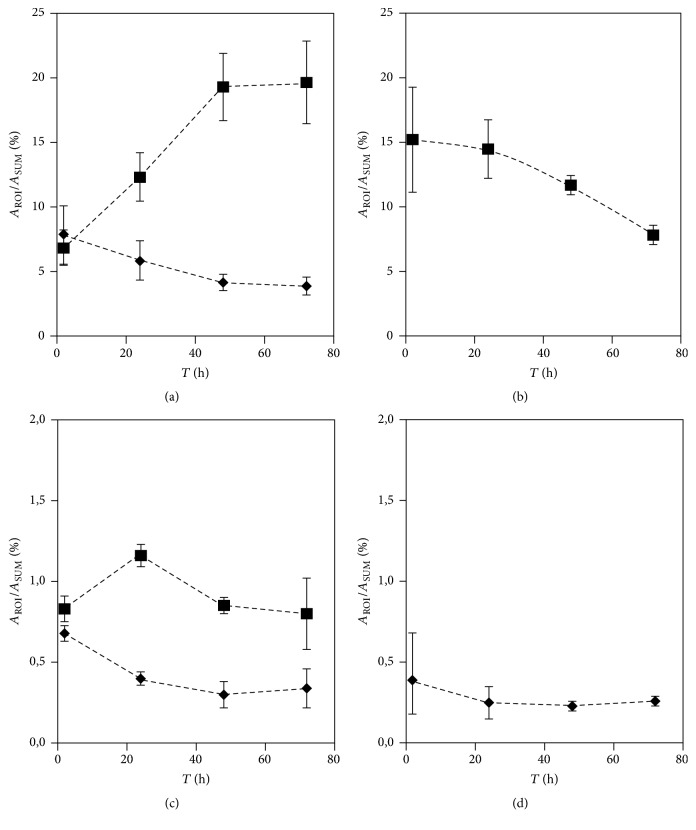
The biodistribution of ^201^Tl doped PBNPs in (a) intestines (square) and liver (diamond), (b) kidneys (square); (c) salivary glands (square) and heart (diamond); and (d) lungs (diamond) in 2, 24, 48, and 72 hours after injection. Brain uptake constantly remained under the threshold level. The mice were under isoflurane anesthesia during the 30 minutes long SPECT scans. The injected activity was 21.07 ± 2.38 MBq.

Activity originating from ^201^Tl-radiolabeled (doped) PBNPs was detected 2 hours after injection in the* liver* (7.89 ± 2.25% ID) and in the* intestines* (6.88 ± 1.37% ID). In later time points (24 hours, 48 hours, and 72 hours) activity in the liver gradually decreased to 5.92 ± 1.78%, 4.17 ± 0.43%, and 4.11 ± 0.64%, respectively. Meanwhile, the activity in the intestines increased to 12.34 ± 1.67%, 19.25 ± 2.32%, and 19.48 ± 3.02%, respectively (Figures 5 and 6(a)).

In the* kidneys *15.24 ± 4.06% of the injected activity was recovered 2 hours after the administration of ^201^Tl doped PBNPs, gradually decreasing to 8.45 ± 0.38% by 72 hours after injection (Figures 5 and 6(b)).

In the* salivary glands* relatively high (0.83 ± 0.08%) activity of ^201^Tl doped PBNPs was observed 2 hours after injection. By 24 hours after injection a statistically significant increase in salivary gland uptake was observed (1.27 ± 0.21%, *p* < 0.05). By 48 and 72 hours after injection activity measured in the salivary glands decreased to 0.78 ± 0.05% and 0.82 ± 0.58%, respectively (Figure 6(c)).

In the* heart ventricles* constantly decreasing activity was observed in every time point when compared to the initial value of 0.59 ± 0.08% (Figures 5 and 6(c)). This is contrary to the usually observed free ^201^Tl ion uptake by heart muscles due to both the very small accumulation and the constant decrease.

In the* lungs* the level of ^201^Tl doped PBNPs was 0.39 ± 0.29% 2 hours after injection, slightly decreasing with time to 0.29 ± 0.05% at 24 hours, 0.27 ± 0.08% at 48 hours, and 0.30 ± 0.06% at 72 hours (Figure 6(d)).

In the* brain* negligable PBNP uptake was recorded 2 hours after injection which did not increase significantly in the next 70 hours (not illustrated).

#### 3.2.2. MRI Measurements

T2-weighted Fast Spin Echo and T1-weighted gradient echo images were acquired to visually evaluate the in vivo biodistribution of PBNP. No signal changes were registered under the applied conditions compared to the prescans.

## 4. Discussion

PB has long been used as a treatment agent of radioactive ^137^Cs poisoning in human medicine based on its successful application in the accident of Goiânia, Brazil [11]. It easily adsorbs Cs^+^ and thus facilitating its excretion from the human body. PB has been registered for the above indication by Food and Drug Administration (FDA) and European Medicines Agency (EMA) as well.

AFM is one of the foremost tools for imaging, measuring, and manipulating subnanometer samples [12]. It measures the shape of nonhydrated particles. The exact diameter of NPs was difficult to determine by AFM due to the tip convolution which leads to artificially increased lateral dimensions on AFM images [13]. Only the height of the particles should be taken into account because the measured width is influenced by tip convolution. The measured height by AFM supported the results of TEM measurements which describe the shape of nonhydrated particles. In both cases the particles appeared as flat rectangular objects which represent the real geometry of the particles. Furthermore, the size distribution of PBNP showed monomodal distribution in cases of TEM and AFM measurements, too.

The average diameter of our PBNPs measured by DLS was similar to the size range reported by Shokouhimehr et al. [4]. These measured hydrodynamic diameters of particles with the small PDI values represent a monodisperse and stable nanobased system in the investigated time frame.

According to the homogeneity and colloidal stability results of PBNPs, the measurements of their other properties were constant. The thermodynamic stability of PB ensured that the synthesized PBNPs could fulfill requirements of long stability as well.

Based on the claims found in the present study authors' 2012 US patent [10], Gallium incorporating PBNP (Ga-PBNP) production studies were performed at the University of Ohio [14]. The resulting structurally very similar Ga-PBNPs to the Tl-PBNPs reported in our study have been throughly characterized. They were also found to be cubic shaped as shown in our results. The size differences could be attributed to the different surface-capping agent (polyvinyl-pyrrolidone).

Citric acid as surface-capping agent to control size and biocompatibility and to avoid agglomeration of the synthetized particles is able to chelate Ca^2+^ ions [15]. However, physiological Ca^2+^ concentration does not affect in vivo colloidal properties [16]. At physiological pH of 7.4, the zeta potential of PBNPs and the steric stabilization effect of citrate coating further enhance the colloidal stability.

MRI T1- and T2-weighted scans showed a more significant T1 shortening effect for PBNPs than T2 shortening. Fe^2+^ in the PB structure is carbon-bound and has a low-spin (*S* = 0), while the nitrogen-bound Fe^3+^ has high spin (*S* = 5/2). Thus, the compound is able to alter both the longitudinal and transverse relaxation times of protons in water molecules [4]. Based on the longitudinal and transversal relaxivity results, a local concentration in the range of 0.5 mM of the ^201^Tl doped PBNPs is needed to achieve proper MRI signal enhancement.

In the case of in vivo MRI scans, we were not able to register any contrast changes between the pre- and postinjection scans. Presumably the iron content of the injected PBNP sample (2.5 mM 250 uL PBNP) was not high enough after the intravenous administration to alter the microscopic magnetic properties of the living system. Further chemical improvement is needed in the future which can enhance the iron (paramagnetic agent) content of the NP or alter the relaxivity of our contrast material. The newly developed iron-based contrast material should be a reliably applicable multimodal contrast agent for in vivo biodistribution studies.

In vivo SPECT scans (based on heart and blood biodistribution measurements) seem to justify that we have attained our main goal of long-term in vivo stability of the labeled PB particles.

No sign of ^201^Tl accumulation in the heart muscles points to any significant in vivo release of free ^201^Tl ions from the particles. As a “biological quality control,” no physiological cardiomyocyte uptake of that free ^201^Tl (which is a potassium ion analogue) is measured.

According to Kevin et al. the in vivo behavior of NPs is mostly regulated by their characteristics such as size, shape, composition, surface chemistry, and associated physical properties [17]. In accordance with the colloidal stability of PBNPs determined by DLS and zeta potential measurements, they were stable in vivo and they did not aggregate in a biologically relevant manner.

The reported citrate-coated PBNPs accumulate in the liver followed by biliary excretion into the intestines. The measured activity in the liver gradually decreased, while the activity in the intestines increased during the 72-hour time period. Uptake mechanisms by Kuppfer cells favor larger, negatively charged particles and are responsible for the retention of most NPs in the liver. Other cell types (liver sinusoidal endothelial cells (LSEC), hepatic stellate cells (HSC), hepatocytes, cholangiocytes, and resident immune cells) also interact with NPs. Uptake by hepatocytes favors smaller and more positively charged particles and leads to their hepatobiliary clearance [18]. Based on the available literature, the size of our PBNPs suggests they were able to extravasate into the spaces of Disse where they were most likely taken up directly by hepatocytes. Kupffer cells and LSECs uptake could have also taken place based on the measured zeta potential. The assumption of Disse-space extravasation and hepatocyte uptake could be further investigated with ultrastructural methods as it represents a targeting opportunity.

Histological examination of previous studies about the biodistribution of NPs provides imporant insight into the routes of accumulation of PBNPs inside the kidneys [19–21]. Based on these experiments, filtration of PBNPs is not possible due to their negative charge and large size; larger PBNPs deposit in the mesangium while smaller NPs remain inside the peritubular vessels.

Kaiser et al. published that salivary glands took up some hydrophilic and negatively charged, cubic shaped NPs already one hour after the i.v. administration [22]. With regard to early tissue distribution pattern of NPs (in our study 2 hours after injection), our results were similar to those of other research groups [17]. The accumulation of our ^201^Tl doped PBNPs in the salivary glands was continuous in the first 24 hours due to the negative surface charge of PBNPs (negative zeta potential) and the abundance of sodium hydrogen carbonate (NaHCO_3_) in the salivary glands which are essencial for entrapping the injected PBNPs. 48 hours after injection the activity detected in the salivary glands decreased most probably due to the excretion of PBNPs into the saliva. Activity levels in the salivary glands decreased and in parallel activity recovered from the intestines continued to increase due to continuous hepatic excretion.

The heart, skeletal muscles, and lungs are not supposed to accumulate PBNPs. The very small radioactivity detected in these organs could be the result of a minuscule proportion of ^201^Tl ions probably released by PBNPs in the bloodstream, where just like in the liver and in the intestines the activity is decreasing.

Because of the radioactive metal binding capability of PBNPs (e.g., ^201^Tl-labeled PBNP), it could be a useful SPECT tracer in preclinical research (in biliary obstructive diseases (e.g., hepatocarcinoma, pancreatic tumors) due to the high and intensive liver uptake and biliary excretion [18]) and could have the potential for translation into clinical practice too. ^201^Tl could be substituted with other radiometals that similarly could be doping the PB structure to ^201^Tl, for example, the beta emitters ^64^Cu, ^90^Sr, ^90^Y, and ^161^Tb and the alpha emitters ^149^Tb and ^213^Bi.

Altogether further development of our NP platform for dual diagnostic and therapeutic functions (theranostics) is of great interest [1]; appropriately modified PBNPs could fulfill the above-mentioned criteria and be useful for the diagnosis and therapy of various diseases including the local radiotherapy of cancer.

## 5. Conclusions

In this study, we successfully synthesized, characterized, and demonstrated the biodistribution of citrate-coated Prussian blue nanoparticles labeled using ^201^Tl isotope. The results show a chemically stable and biocompatible ^201^Tl-labeled nanoparticulate SPECT tracer. Important hepatic and salivary glands uptake was seen to be of particular interest upon evaluation of the particle biodistribution. These PB-based particles could be applied as a drug delivery platform or a contrast agent in preclinical research. They could further be tailored towards clinical application, too.

## Figures and Tables

**Table 1:** Result of relaxivities (*r*1 and *r*2), ordered to examine concentrations of ^201^Tl doped PBNPs.

Relaxivity (mM^−1^ms^−1^)	*R* (mM^−1^ms^−1^)	Error of *R* (mM^−1^ms^−1^)
*r*1 = 0.64+/−0.02	3089	91
*r*2 = 0.96+/−0.03	2119	350

**Table 2:** Retention factors of ^201^Tl(I) ions and ^201^Tl doped PBNP examined with paper chromatography.

Sample	Retention factor	Activity
Standard [^201^Tl]TlCl_aq_	0.85	86.8%
^201^Tl doped PBNP	0.02	85.2%

## Supplementary Materials

Supplementary Materials Table S1: the result of viability assays in cases of saline and three different PBNP dilutions. After 20-minute incubation period intact and dead HeLa cells were counted by each dilution. Cell morphology of dead cells from viable ones was distinguished by shape, light reflection, and scattering properties and lack of motion.
